# Screening of three-way crossbred combination and genetic effect analysis of the SNP in the *CLPG* gene in meat sheep

**DOI:** 10.5194/aab-65-417-2022

**Published:** 2022-11-17

**Authors:** Jian Tao Wang, Guo Sen Wang, Yuan Fang Gong, Xian Qiao, Xiang Li, Gui Zhu Wang, Ying Zhen Zheng, Jian Guo Lv, Xiang Long Li, Zheng Zhu Liu

**Affiliations:** 1 Breeding Livestock and Poultry Management Department, Tangshan Animal Husbandry Technology Promotion Station, Tangshan 063000, China; 2 Hebei Key Laboratory of Specialty Animal Germplasm Resources Exploration and Innovation, College of Animal Science and Technology, Hebei Normal University of Science & Technology, Qinhuangdao 066004, China; 3 Tianjin DBN Sci-tech Group, Chang Nong Aquatic Science and Technology Co. Ltd, Tianjin 301804, China

## Abstract

In order to promote the rapid development of the meat sheep industry, a three-way crossbred combination experiment was carried out with Australian White, Dorper, and Charollais sheep as terminal male parents and the elite F1 hybrids of Australian White × Small-tailed Han (Han), Dorper × Han, and Charollais × Han as female parents, which was based on the screening of a two-way crossbred combination in meat sheep. The growth performance of six groups of three-way crossbred combinations and Han lambs was measured and analyzed, and the effect of a polymorphism in the *CLPG* gene on the growth performance of three-way crossbred lambs was also studied. The results showed that under the same rearing conditions, weight at 3 and 6 months of age and average daily gain from birth to 3 months and from 3 to 6 months of age were all the largest for Australian × (Charollais × Han) crossbred lambs. They were significantly or extremely significant different from the other three-way crossbred combinations and Han lambs ( P<0.05 , P<0.01 ). The body height, body length, chest girth, and cannon bone circumference at 3 months of age and body length, chest girth, and cannon bone circumference at 6 months of age were also the largest for Australian × (Charollais × Han) crossbred lambs. Among them, body length, chest girth, and cannon bone circumference at 3 months of age were significantly different from the other three-way crossbred combinations and Han lambs ( P<0.05 ), and body length, chest girth, and cannon bone circumference at 6 months of age were significantly or extremely significant different from the other three-way crossbred combinations and Han lambs ( P<0.05 , P<0.01 ). The potential genetic effects of the *CLPG* gene on the growth performance indicators of three-way crossbred lambs showed that a mutation site ( g .232 C > T ) of this gene had two genotypes: *CC* and *CT*. Among them, the data of body weights and body sizes from *CT* genotype individuals at birth, 3 months old, and 6 months old were significantly higher than those of *CC* genotype individuals, and some indicators showed significant or extremely significant differences ( P<0.05 , P<0.01 ), suggesting that higher growth performance was observed in individuals with T alleles. To sum up, the crossbred combination of Australian × (Charollais × Han) could be suggested as the optimal choice. The T allele of the *CLPG* gene showed potential advantages in the performance of meat production in meat sheep. Based on the current results, we recommend that the offspring of Australian × (Charollais × Han) with the T allele should be preferentially utilized for meat sheep production.

## 1 Introduction

Mutton is a kind of healthcare food that is rich in nutrients such as proteins and low in fat and cholesterol. With the continuous improvement of per capita income and awareness of living a healthy life, mutton consumption has increased over the years, which has greatly promoted the rapid development of the meat sheep industry in China (Li et al., 2019; Tang et al., 2016). However, due to the lack of predominant meat sheep breeds, and the absence of a well-established meat sheep crossbreeding system, as well as poor farming management, mutton production in China has been far from meeting the needs of the domestic market in recent years. Many studies have shown that the use of hybrid advantages by two-way or three-way crossbreeding can greatly improve the meat production of sheep (Sun et al., 2017; Wang et al., 2015; Rehemanet al., 2015). At present, the production of commercialized meat sheep mostly adopts a two-way crossbreeding model, in which local sheep breeds with good reproductive performance, high adaptability, and large body size are used as maternal parents, and imported high-quality meat sheep breeds are used as male parents, and the hybrid offspring are slaughtered for sale. On this basis, three-way crosses can be performed with a third excellent meat breed of male sheep as the terminal parent, and the offspring are fattened and sold. Three-way crosses bring together the advantages of maternal and individual heterosis (Yang et al., 2015), and this method has been widely used in pigs and sheep. The results show that the growth performance of three-way crossbred lambs is superior to two-way crossbred lambs and local breeds (female parents) (Song et al., 2009; Wang et al., 2018; Du et al., 2016).

The *CLPG* gene is a functional gene that affects animal muscle growth and meat tenderness (Chen et al., 2011; Jawasreh et al., 2019; Moriah et al., 2017), and mutation in this gene can lead to muscle hypertrophy in the waist, buttock, and limbs of sheep (Cockett et al., 2005; Jackson et al., 1997; Koohmaraie et al., 1995). To improve the meat production of Awassi sheep, Jawasreh et al. (2019) performed artificial insemination with frozen semen from a male Rambouillet with a homozygous *CLPG* mutation; afterwards, fattened lambs showed improvement in body weight, average daily gain, carcass weight, and net meat weight compared to Awassi sheep (Jawasreh et al., 2019). Significantly lower back fat thickness ( P<0.05 ) and greater eye muscle area ( P<0.01 ) of individuals in *CT* genotype with a C→T ( g .232 C > T ) mutation in *CLPG* than in *CC* genotype individuals of Australian White × (Dorper × Hu) sheep were reported by Hu et al. (2016). Based on the screening of two-way cross hybrids of meat sheep (Liu et al., 2020), we conducted three-way crosses to analyze the growth performance of three-way cross hybrids and Small-tailed Han (Han) lambs. We also investigated the function of the single-nucleotide polymorphism (SNP) in *CLPG* in three-way crossbred lambs. The aim of this study is to select the best three-way crossbred combinations from phenotypic and molecular levels and to provide basic materials for local meat sheep production.

## 2 Materials and methods

### 2.1 Growth performance of three-way crossbred lambs

#### 2.1.1 Experimental site and cross combinations

On the basis of selected two-way cross combinations of meat sheep, three-way crosses were performed with Australian White, Dorper, and Charollais as terminal male parents and Australian White × Han, Dorper × Han, and Charollais × Han F1 crossbred lambs as female parents. A total of six combinations were obtained, including Australian × (Dorper × Han) (ADH), Australian × (Charollais × Han) (ACH), Dorper × (Australian × Han) (DAH), Dorper × (Charollais × Han) (DCH), Charollais × (Australian × Han) (CAH), and Charollais × (Dorper × Han) (CDH). Purebred Han lambs were used as control. All lambs were weaned at 3 months of age and slaughtered at 6 months of age using the same rearing management techniques.

#### 2.1.2 Determination of growth performance

A total of 76 three-way crossbred lambs and Han lambs were obtained, including seven groups (10 ADH, 14 ACH, 10 DAH, 10 DCH, 4 CAH, 10 CDH, and 18 Han lambs as control). Each group had the same number of males and females.

Body weight and body size parameters were measured by an electronic scale and measuring stick according to the literature (Liu et al., 2020). Body weight parameters included birth weight, 3-month-old weight, 6-month-old weight, and average daily gain from birth to 3 months and from 3 to 6 months of age. Body size parameters included body height, body length, chest circumference, and cannon bone circumference.

#### 2.1.3 Data analysis

One-way ANOVA (analysis of variance; SPSS 20.0 software) was used to evaluate the interaction between different groups for growth performance including body weight and body size. The values were expressed as the mean ± standard deviation. And least significant difference (LSD) test was used to compare the differences, considering P<0.05 as the significance level.

### 2.2 Effects of the SNP in the *CLPG* gene in three-way crossbred lambs

#### 2.2.1 Lambs

A total of 76 three-way crossbred lambs and Han lambs were obtained, including 58 three-way crossbred lambs (6 combinations) and 18 Han lambs. From six combinations, 37 three-way crossbred lambs were selected randomly for the study of the effects of the SNP in the *CLPG* gene.

#### 2.2.2 Sample collection and processing

Blood samples were collected from the jugular vein of the sheep, and after the serum naturally precipitated, the blood clot was centrifuged for genomic DNA extraction and processed as described in this paper (Liu et al., 2020).

#### 2.2.3 DNA extraction, primer design, PCR amplification, and product recovery

DNA extraction was performed according to the literature (Liu et al., 2020). The DNA sequence of the sheep *CLPG* gene including the SNP locus (AF401294.1) was found on GenBank, and a pair of primers around this SNP was designed by the online software Primer 3: F 5 ${}^{\prime }$ -ATCATCGTGTCCTGGTCTATTTTCG-3 ${}^{\prime }$ and R 5 ${}^{\prime }$ -TAATGAAAGATTGAGGGGATGTTGG-3 ${}^{\prime }$ . The PCR product was 493 bp in length. After PCR amplification, the PCR product was recovered using the agarose gel DNA recovery kit and sent for sequencing. Primer synthesis and sequencing were completed by Shanghai Bioengineering Technology Service Co., Ltd (Shanghai, China).

#### 2.2.4 PCR-RFLP of the *CLPG* gene

Polymerase chain reaction–restriction fragment length polymorphism (PCR-RFLP) is the most simple method or single-nucleotide change detection. Using the network digestion software (http://www.addgene.org/analyze-sequence/, last access: 7 July 2021), the amplification product of the *CLPG* gene of the ternary hybrid sheep was pre-digested, and we found that the restriction enzyme Hha I had the recognition site of the mutation sequence of the gene. Then the PCR products of all the subjected ternary hybrid sheep were digested by digestion, and the reaction system and reaction conditions are shown in Table A1.

#### 2.2.5 Association analysis of mutation and growth traits

Association analysis was performed to elucidate the effect of the *CLPG* mutation on body weight and body size parameters at 3 and 6 months of age of three-way crossbred lambs using the least-squares means from a general linear model (GLM) in SAS software (version 8.2). The values were expressed as the least-squares mean ± standard error.

$$Y=\mu +s_{i}+g_{j}+tx_{h}+e,$$

where Y is the observational values of growth traits of F1 generation sheep hybridized with different meat sheep, μ is the population mean, $s_{i}$ is the gender fixation effect, $g_{j}$ is the genotype fixation effect, $tx_{h}$ is the birth type fixation effect, and e is the random residual effect.

## 3 Results

### 3.1 Growth performance of three-way crossbred and Han lambs

#### 3.1.1 Body weight

Variance analysis of body weight and average daily gain at different ages (birth, 3 months old, and 6 months old) of 76 three-way crossbred and Han lambs was performed. Differences of body weight parameters between three-way crossbred and Han lambs under the same rearing conditions are presented in Table A2. The birth weight of CDH (4.09 ± 1.06 kg) was the highest, followed by ACH (3.98 ± 0.95 kg), while no significant differences were observed among groups ( P>0.05 ). The 3-month-old weight of ACH (27.83 ± 6.58 kg) was the highest, which was significantly different from Han ( P<0.05 ), whereas no significant differences were observed among the other groups. ACH (44.89 ± 6.54 kg) was significantly different from CAH and CDH ( P<0.05 ) and was highly significantly different from Han ( P<0.01 ). Average daily gain from birth to 3 months of age was highest in ACH (243.32 ± 83.02 g d ${}^{-1}$ ), which was highly significantly different from CAH ( P<0.01 ) and significantly different from Han ( P<0.05 ). Average daily gain from 3 to 6 months of age was highest for ACH (299.57 ± 42.13 g d ${}^{-1}$ ), which was significantly different from CAH, CDH, and Han ( P<0.05 ). Taken together, ACH exhibited optimal body weight.

#### 3.1.2 Body size

Variance analysis was performed on body size parameters of 76 three-way crossbred and Han lambs at 3 and 6 months of age under the same rearing conditions (Tables A3 and A4). Body size parameters of ACH at 3 months of age were the highest in all groups, as shown in Table A3. Body height of ACH (59.89 ± 4.97 cm) was significantly different from DAH and CDH ( P<0.05 ). Body length and chest circumference of ACH (65.00 ± 6.21 cm and 75.76 ± 5.98 cm, respectively) were significantly differed from ADH, DAH, and Han ( P<0.05 ). Cannon bone circumference of ACH (7.94 ± 0.45 cm) was significantly different from ADH, DAH, CAH, and Han ( P<0.05 ). The results indicate that ACH had optimal body size parameters at the age of 6 months, as shown in Table A4. Body height was highest for Han lambs (68.16 ± 3.72 cm), followed by CAH (67.82 ± 3.54 cm) and ACH (67.39 ± 2.12 cm), whereas no significant differences were observed among groups ( P>0.05 ). Body length was highest in ACH (76.62 ± 3.17 cm), which was significantly different from ADH and Han ( P<0.05 ). Chest circumference was highest in ACH (86.90 ± 4.13 cm), which was significantly different from ADH and DAH ( P<0.05 ) and was highly significantly different from Han ( P<0.01 ). Cannon bone circumference was highest in ACH (10.07 ± 0.51 cm) and significantly different from Han ( P<0.05 ). In sum, ACH showed optimal body size parameters at the age of 3 and 6 months.

### 3.2 Effects of the SNP in *CLPG* in three-way crossbred lambs

#### 3.2.1 PCR amplification of *CLPG*

**Figure B1:**
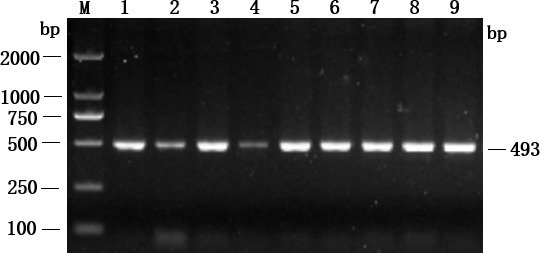
Detection of *CLPG* PCR products from three-way crossbred lambs (M, DL2000; 1–9, PCR products).

PCR amplification was successful for the 37 three-way crossbred lambs, and the results are shown in Fig. B1. A bright and clear band with an approximate length of 500 bp was observed, which coincided with the expected product length of 493 bp.

#### 3.2.2 Restriction endonuclease digestion of *CLPG*

**Figure B2:**
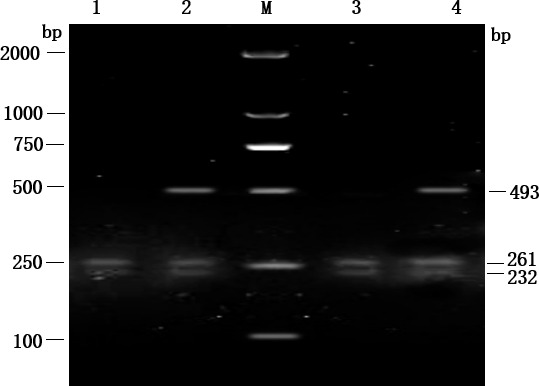
Digestion of *CLPG* PCR products from three-way crossbred lambs (M, DL2000; 1–4, digestion products; 1 and 3, *CC* individuals; 2 and 4, *CT* individuals).

The amplified products (493 bp) of *CLPG* from three-way crossbred lambs were digested with Hha I at 232 bp. As shown in Fig. B2, two genotypes were identified, *CC* (232 and 261 bp) and *CT* (493, 232, and 261 bp).

#### 3.2.3 Association analysis of the SNP in *CLPG* and growth traits

Least-squares analysis of the body weight and body size parameters of three-way crossbred lambs with different *CLPG* genotypes of g .232 C > T is shown in Tables A5 and A6. After sequencing, 25 *CT* genotypes and 12 *CC* genotypes were detected. According to Table A5, comparing *CT* with *CC* genotypes, we found that there were increases in birth weight (32.31 %), weight at 3 months of age (13.60 %), and weight at 6 months of age (14.41 %). There were significant differences in birth weight and weight at 6 months of age between the two genotypes ( P<0.05 ).

As Table A6 shows that body length, body height, chest circumference, and cannon bone circumference at 3 and 6 months of age were higher in the *CT* genotype than the *CC* genotype. Based on the data from Table A6, the increases were 6.83 %, 10.13 %, 3.13 %, and 4.02 % at 3 months of age and 3.96 %, 3.88 %, 3.91 %, and 15.50 % at 6 months of age, respectively. There was a significant difference in cannon bone circumference at 6 months of age between the *CT* and *CC* genotype ( P<0.05 ).

## 4 Discussion

### 4.1 Growth performance of three-way crossbred and Han lambs

Han sheep is one of the best local breeds in China, famous for rapid growth and development, early sexual maturity, prolificacy, and high body height, as well as estrous cycles throughout the year (Zhao, 2011; China Committee on Animal Genetic Resources, 2011; Qu el., 2003). Australian White, Dorper, and Charollais are also the best meat sheep breeds globally, with merits of rapid growth and development, high meat production, and tender and juicy meat (Liu et al., 2020; Zhao, 2011; China Committee on Animal Genetic Resources, 2011). In this research, we studied three-way crossbred lambs, including ADH, ACH, DAH, DCH, CAH, and CDH, together with Han lambs as control. We found that under the same rearing conditions, the birth weight of ACH was slightly lower than CDH but higher than the other five combinations, and there were no significant differences among groups ( P>0.05 ). The body height of ACH at the age of 6 months was slightly lower than Han and CAH but higher than the other four combinations, and there were no significant differences among groups. In addition, ACH was superior to other combinations and Han lambs in terms of body weight and body size at 3 and 6 months of age and average daily gain from birth to 3 months and from 3 to 6 months of age. These results suggest that ACH crossbred lambs with Australian White as terminal male parents and Charollais × Han as female parents have more advantages in production performance, which is consistent with previous studies (Song et al., 2009; Zhao et al., 2018; Zhao et al., 2018). Zhao et al. (2018) found that compared with purebred Han, the F1 hybrids of Texel × Dorset × Han had significantly higher birth weight and weight at 3 and 6 months of age and average daily gain at 3 and 6 months ( P<0.05 ) (Zhao et al., 2018). The previous results of Zhao et al. (2018) showed that purebred Han had significantly lower birth weight and weight at 1 to 6 months of age compared to three-way crossbred lambs, including polled Dorset × White Suffolk × Han, Texel × White Suffolk × Han, polled Dorset × German White × Han, and Texel × polled Dorset × Han ( P<0.05 ) (Zhao et al., 2018). Song et al. (2009) also found that purebred Han had highly significantly lower birth weight and weight at 3 and 6 months of age compared to three-way crossbred combinations like polled Dorset × Romney × Han and polled Dorset × Dorset × Han ( P<0.01 ) (Song et al., 2009). Australian White sheep is the first sheep breed developed using modern genetic testing techniques in Australia that carries the excellent genes of White Dorper, Van Rooy, polled Dorset, and Texel (Freking et al., 2002). Our results provide strong evidence that this breed could improve the growth performance including high weight, large size, and rapid growth traits to its offspring when used as a terminal male parent in three-way crossbreeding for commercial production (Freking et al., 2002).

### 4.2 Effects of the SNP in *CLPG*

Previous studies have shown that *CLPG* mutations on ovine chromosome 18 can lead to muscle hypertrophy in sheep (Moriah et al., 2017; Cockett et al., 2005; Jackson et al., 1997; Freking et al., 2002; Smit et al., 2003; Freking et al., 2018). *CLPG* is known to play a role in altering the expression of delta-like homolog 1 (DLK1), a member of the epidermal growth factor (EGF)-like family of homeotic proteins involved with cell–cell communication related to adipocyte differentiation and muscle development (White et al, 2008; Bidwell et al., 2014). In this study, we analyzed the effects of *CLPG* mutation g .232 C > T on the growth of three-way crossbred lambs – ADH, ACH, DAH, DCH, CAH, and CDH. Individuals with the *CT* genotype were superior to those with the *CC* genotype in terms of body weight and body size parameters from birth to 6 months of age. This result is consistent with the findings of our colleagues Liu et al. (2020) on two-way crossbred F1 offspring of Australian × Han, Dorper × Han, and Charollais × Han (Liu et al., 2020). And more similar results were found in the research of Hu et al. (2016) and Zhang et al. (2014). These studies identified the same mutation site on *CLPG* as shown in the present study. Liu et al. (2020) found that the T allele promotes the growth and development of F1 hybrids of Australian × Han, Dorper × Han, and Charollais × Han (Liu et al., 2020). Hu et al. (2016) showed that this mutation was significantly or highly significantly correlated with backfat thickness and eye muscle area in Australian White × (Dorper × Hu) ( P<0.05 , P<0.01 ), and the T allele facilitates fat loss and muscle growth (Hu et al., 2016). Zhang et al. (2014) reported that female Australian White sheep with T at this site had significantly or highly significantly improved body weight and body length ( P<0.05 , P<0.01 ) (Zhang et al., 2014). Freking et al. (2018) found that differences in birth weight were detected ( P<0.01 ) for the combination of the two loci from *MSTN* and *CLPG* and birth type, with single-born differences among genotypes exceeding differences among twin born progeny (Freking et al., 2018). The results indicate that differences in birth weight of our result might be caused by genotype combinations influenced while interacting with birth type. Our study confirmed that the T allele of g .232 C > T facilitated the growth of meat sheep; however, more experiments are needed for further validation in the future.

## 5 Conclusions

In this study, a three-way crossbred combination experiment was performed with Australian White, Dorper, and Charollais as terminal male parents and elite F1 hybrids of Australian × Han, Dorper × Han, and Charollais × Han as female parents. According to the growth performance of 6 groups of three-way crossbred sheep and Han lambs as well as the potential effects of *CLPG*, the offspring of male Australian White and female Charollais × Han showed apparent performance advantages, and the individuals with the T allele had improved body weight and body size. We suggest that the offspring of ACH harboring the T allele in *CLPG* should be preferentially utilized for meat sheep production in Tangshan, Hebei Province.

## Data Availability

The data that support this study cannot be publicly shared due to ethical or privacy reasons and may be shared upon reasonable request to the corresponding author if appropriate.

## Appendix A

**Table A1:** System components and reaction conditions for enzyme digestion of *CLPG* PCR products.

Reaction system		Reaction conditions	
PCR products	3.0 µ L	Temperature	37 ${}^{\circ }$ C
10 × Buffer	4.0 µ L	Duration	1.5 h
ddH ${}_{2}$ O	8.2 µ L		
Hha I	1.0 µ L		

**Table A2:** The result of variance analysis of body weight parameters of three-way crossbred and Han lambs (mean ± standard deviation).

Body weight	ADH	ACH	DAH	DCH	CAH	CDH	Han
(kg)	( n = 10)	( n = 14)	( n = 10)	( n = 10)	( n = 4)	( n = 10)	( n = 18)
Birth	3.66 ± 0.86 ${}^{a}$	3.98 ± 0.95 ${}^{a}$	3.41 ± 0.82 ${}^{a}$	3.72 ± 0.76 ${}^{a}$	3.50 ± 0.71 ${}^{a}$	4.09 ± 1.06 ${}^{a}$	3.77 ± 0.91 ${}^{a}$
At 3 months of age	25.74 ± 5.84 ${}^{\mathrm{ab}}$	27.83 ± 6.58 ${}^{a}$	24.75 ± 1.58 ${}^{\mathrm{ab}}$	22.49 ± 4.44 ${}^{\mathrm{ab}}$	27.73 ± 1.34 ${}^{\mathrm{ab}}$	25.62 ± 5.27 ${}^{\mathrm{ab}}$	22.94 ± 3.39 ${}^{b}$
At 6 months of age	42.97 ± 5.98 ${}^{\mathrm{ab}}$	44.89 ± 6.54 ${}^{a}$	42.42 ± 6.54 ${}^{\mathrm{ab}}$	42.31 ± 7.32 ${}^{\mathrm{ab}}$	40.20 ± 0.47 ${}^{\mathrm{bc}}$	40.21 ± 1.65 ${}^{\mathrm{bc}}$	39.62 ± 4.11 ${}^{c}$
Average daily gain from	238.21 ± 64.95 ${}^{a}$	243.32 ± 83.02 ${}^{a}$	239.76 ± 75.83 ${}^{a}$	208.81 ± 73.33 ${}^{a}$	170.23 ± 18.12 ${}^{c}$	224.16 ± 72.24 ${}^{a}$	181.86 ± 75.50 ${}^{\mathrm{bc}}$
birth to 3 months of age							
Average daily gain from	286.35 ± 31.56 ${}^{a}$	299.57 ± 42.13 ${}^{a}$	283.55 ± 49.79 ${}^{a}$	278.64 ± 57.31 ${}^{\mathrm{ab}}$	270.15 ± 68.95 ${}^{b}$	273.82 ± 52.47 ${}^{b}$	268.79 ± 73.68 ${}^{b}$
3 to 6 months of age							

Note that the same letters in a column indicate no significant differences ( P>0.05 ), adjacent letters significant differences ( P<0.05 ), and alternate letters extremely significant differences ( P<0.01 ). The same notation has been used in Tables A3 and 4.

**Table A3:** The result of variance analysis of body size parameters of three-way crossbred and Han lambs at 3 months of age (mean ± standard deviation).

Body size	ADH	ACH	DAH	DCH	CAH	CDH	Han
(cm)	( n = 10)	( n = 14)	( n = 10)	( n = 10)	( n = 4)	( n = 10)	( n = 18)
Body length	59.16 ± 2.91 ${}^{b}$	65.00 ± 6.21 ${}^{a}$	59.66 ± 2.66 ${}^{b}$	63.48 ± 4.54 ${}^{a}$	62.32 ± 6.98 ${}^{\mathrm{ab}}$	62.34 ± 3.96 ${}^{\mathrm{ab}}$	58.51 ± 4.49 ${}^{b}$
Body height	59.01 ± 2.55 ${}^{a}$	59.89 ± 4.97 ${}^{a}$	55.47 ± 3.34 ${}^{b}$	59.18 ± 3.07 ${}^{a}$	58.15 ± 3.04 ${}^{a}$	55.03 ± 3.06 ${}^{b}$	58.74 ± 3.42 ${}^{a}$
Chest circumference	70.96 ± 5.57 ${}^{b}$	75.76 ± 5.98 ${}^{a}$	72.80 ± 3.84 ${}^{b}$	75.26 ± 6.19 ${}^{a}$	73.44 ± 4.16 ${}^{\mathrm{ab}}$	73.77 ± 3.17 ${}^{\mathrm{ab}}$	70.22 ± 5.47 ${}^{b}$
Cannon bone circumference	7.59 ± 0.52 ${}^{b}$	7.94 ± 0.45 ${}^{a}$	7.52 ± 0.66 ${}^{b}$	7.76 ± 0.38 ${}^{a}$	7.54 ± 0.49 ${}^{b}$	7.72 ± 0.52 ${}^{\mathrm{ab}}$	7.33 ± 0.48 ${}^{b}$

**Table A4:** The result of variance analysis of body size parameters of three-way crossbred and Han lambs at 6 months of age (mean ± standard deviation).

Body size	ADH	ACH	DAH	DCH	CAH	CDH	Han
(cm)	( n = 10)	( n = 14)	( n = 10)	( n = 10)	( n = 4)	( n = 10)	( n = 18)
Body length	72.35 ± 2.62 ${}^{b}$	76.62 ± 3.17 ${}^{a}$	73.58 ± 5.15 ${}^{\mathrm{ab}}$	74.65 ± 4.76 ${}^{a}$	74.57 ± 3.94 ${}^{a}$	74.48 ± 4.58 ${}^{a}$	73.24 ± 4.33 ${}^{b}$
Body height	65.06 ± 2.93 ${}^{a}$	67.39 ± 2.12 ${}^{a}$	65.87 ± 3.61 ${}^{a}$	66.87 ± 2.28 ${}^{a}$	67.82 ± 3.54 ${}^{a}$	66.01 ± .96 ${}^{a}$	68.16 ± 3.72 ${}^{a}$
Chest circumference	84.21 ± 4.01 ${}^{\mathrm{bc}}$	86.90 ± 4.13 ${}^{a}$	85.30 ± 2.15 ${}^{\mathrm{bc}}$	86.42 ± 3.31 ${}^{\mathrm{ab}}$	86.40 ± 6.23 ${}^{\mathrm{ab}}$	86.38 ± 2.48 ${}^{\mathrm{ab}}$	84.05 ± 5.94 ${}^{c}$
Cannon bone circumference	9.87 ± 0.75 ${}^{a}$	10.07 ± 0.51 ${}^{a}$	9.79 ± 0.78 ${}^{a}$	9.89 ± 0.48 ${}^{a}$	9.88 ± 0.61 ${}^{a}$	9.80 ± 046 ${}^{a}$	9.58 ± 0.49 ${}^{b}$

**Table A5:** Least-squares analysis of body weight parameters of three-way crossbred lambs with different genotypes (least-squares mean ± standard error).

Body weight (kg)	*CC* genotype	*CT* genotype
Birth	3.22 ± 0.34 ${}^{b}$	4.27 ± 0.33 ${}^{a}$
At 3 months of age	22.55 ± 1.63	25.54 ± 1.57
At 6 months of age	40.25 ± 1.52 ${}^{b}$	46.05 ± 1.48 ${}^{a}$

Note that the absence of letters indicates no significant differences ( P>0.05 ), and different letters indicate significant differences ( P<0.05 ). The same notation is employed in Table A6.

**Table A6:** Least-squares analysis of body size parameters of three-way crossbred lambs with different genotypes at 3 and 6 months of age (least-squares mean ± standard error).

Body size (cm)	At 3 months of age		At 6 months of age	
	*CC* genotype	*CT* genotype	CC genotype	*CT* genotype
Body length	52.35 ± 1.97	55.93 ± 1.90	72.08 ± 1.27	74.93 ± 1.23
Body height	53.10 ± 1.26	58.48 ± 1.23	66.57 ± 1.10	69.15 ± 1.07
Chest circumference	70.06 ± 0.06	72.25 ± 2.07	83.93 ± 1.69	87.21 ± 1.63
Cannon bone circumference	7.47 ± 0.20	7.77 ± 0.19	9.03 ± 0.15 ${}^{b}$	10.43 ± 0.15 ${}^{a}$
